# 2-[(*E*)-2-(Benzyl­idene­amino)­eth­yl]-3′,6′-bis­(diethyl­amino)­spiro­[isoindoline-1,9′-xanthen]-3-one

**DOI:** 10.1107/S1600536811020320

**Published:** 2011-06-18

**Authors:** Zhen Wei, Xujun Zheng, Junjun Bai, Xiaohong Zhai, Lili Song

**Affiliations:** a Department of Basic Science, Tianjin Agriculturial College, Tianjin Jinjing Road No. 22, Tianjin 300384, People’s Republic of China

## Abstract

In the title compound, C_37_H_40_N_4_O_2_, the xanthene and spiro­lactam rings are almost planar, with r.m.s. deviations from the mean planes of 0.223 (2) and 0.057 (2) Å, respectively, and form a dihedral angle of 85.76 (3)°. The dihedral angle between the xanthene mean plane and the benzene ring is 87.16 (5)°. One of the two ethyl groups of one of the diethyl­amino groups is disordered over two sets of sites [0.76 (1):0.24 (1)].

## Related literature

For related structures and background to rhodamine dyes, see: Xu *et al.* (2010*a* ▶,*b* ▶); Zhang *et al.* (2008 ▶); Tian *et al.* (2008 ▶); Kwon *et al.* (2005 ▶); Wu *et al.* (2007 ▶).
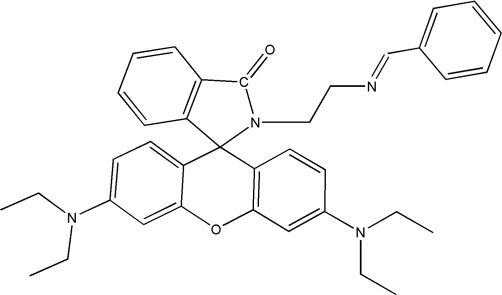


## Experimental

### 

#### Crystal data

C_37_H_40_N_4_O_2_
*M* *_r_* = 572.73
Triclinic, 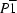

*a* = 9.842 (2) Å
*b* = 13.151 (3) Å
*c* = 13.552 (3) Å
α = 74.43 (3)°
β = 81.92 (3)°
γ = 69.12 (3)°
*V* = 1576.7 (7) Å^3^
*Z* = 2
Mo *K*α radiation
μ = 0.08 mm^−1^
*T* = 293 K
0.26 × 0.22 × 0.20 mm

#### Data collection

Rigaku Saturn diffractometer
Absorption correction: multi-scan (*CrystalClear*; Rigaku, 2008 ▶) *T*_min_ = 0.981, *T*_max_ = 0.985
17336 measured reflections
6168 independent reflections
4273 reflections with *I* > 2σ(*I*)
*R*_int_ = 0.033

#### Refinement

*R*[*F* ^2^ > 2σ(*F* ^2^)] = 0.057
*wR*(*F* ^2^) = 0.174
*S* = 1.06
6168 reflections
399 parameters
2 restraints
H-atom parameters constrained
Δρ_max_ = 0.29 e Å^−3^
Δρ_min_ = −0.24 e Å^−3^

### 

Data collection: *CrystalClear* (Rigaku, 2008 ▶); cell refinement: *CrystalClear*; data reduction: *CrystalClear*; program(s) used to solve structure: *SHELXS97* (Sheldrick, 2008 ▶); program(s) used to refine structure: *SHELXL97* (Sheldrick, 2008 ▶); molecular graphics: *SHELXTL* (Sheldrick, 2008 ▶); software used to prepare material for publication: *SHELXL97*.

## Supplementary Material

Crystal structure: contains datablock(s) I, global. DOI: 10.1107/S1600536811020320/bg2398sup1.cif

Structure factors: contains datablock(s) I. DOI: 10.1107/S1600536811020320/bg2398Isup2.hkl

Supplementary material file. DOI: 10.1107/S1600536811020320/bg2398Isup3.cdx

Supplementary material file. DOI: 10.1107/S1600536811020320/bg2398Isup4.cml

Additional supplementary materials:  crystallographic information; 3D view; checkCIF report

## Figures and Tables

**Fig. 1.**
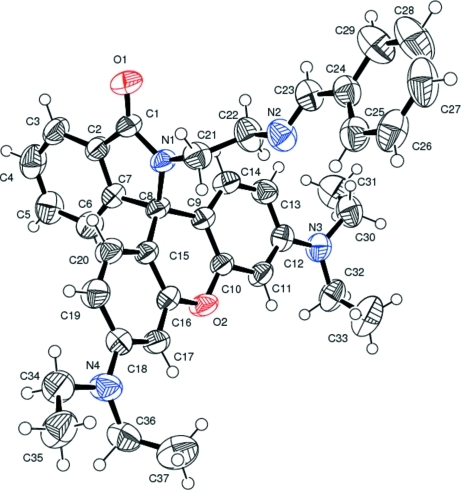
The molecular structure of the title compound with the atom numbering scheme. Displacement ellipsoids are drawn at the 30% probability level. Only the major part of the ethyl group attached to N4 has been drawn, for clarity.

## supplementary crystallographic information

### Comment

Rhodamine dyes are known to have excellent photophysical properties,and they are one of the most widely used fluorophores for labeling and sensing biomolecules. There are a few single-crystal reports on rhodamine derivatives bearing a lactam moiety (Xu *et al.*, 2010*a*;2010*b* Kwon *et al.*, 2005; Wu *et al.*, 2007; Zhang *et al.*, 2008; Tian *et al.*, 2008). Detailed information on their molecular and crystal structures is necessary to understand their photophysical and photochemical properties.

In the title compound, C_37_H_20_N_4_O_2_, the xanthene and spirolactam-rings are almost planar, with r.m.s. deviations from the mean planes of 0.223 (2)Å and 0.057 (2)Å, respectively, and form a dihedral angle of 85.76 (3)°. The dihedral angle between the xanthene mean plane and the benzene ring is 87.16 (5)°. During refinement, one of the two ethyl groups bonded to N4 appeared disordered. The corresponding occupancies refined to final values of 0.76/0.24 (1).

### Experimental

*N*-(rhodamine-6 G)lactam-ethylenediamine (10*m* mol) was dissolved in 20 ml of ethanol, followed by addition of benzaldehyde(15*m* mol). The solution was stirred and refluxed for 4 h when white precipitate appeared,the resulting crude product was obtained by filteration. then the product was disolved in ethanol,Single crystals suitable for X-ray measurements were obtained from ethanol by slow evaporation at room temperature.

### Refinement

During refinement, one of the two ethyl groups bonded to N4 appeared disordered. The corresponding occupancies refined to final values of 0.76/0.24 (1), and were kept fixed afterwards. The disordered model was refined using the tools availablein *SHELXL97* (Sheldrick, 2008): SADI for restraining distances, EADP to correlate anisotropic thermal parameters for related disordered atoms.

All H atoms were geometrically positioned and treated as riding on their parent atoms, with C—H = 0.93 Å for the aromatic, 0.96 Å for the methyl H atoms and C—H= 0.97 Å for methylene, with *U*_iso_(H)= 1.2 *U*_eq_(C aromatic) or,1.5*U*_eq_(C methyl).

### Crystal data

**Table:**

C_37_H_40_N_4_O_2_	*Z* = 2
*M**_r_* = 572.73	*F*(000) = 612
Triclinic, *P*1	*D*_x_ = 1.206 Mg m^−^^3^
Hall symbol: -P 1	Mo *K*α radiation, λ = 0.71073 Å
*a* = 9.842 (2) Å	Cell parameters from 3876 reflections
*b* = 13.151 (3) Å	θ = 1.6–29.0°
*c* = 13.552 (3) Å	µ = 0.08 mm^−^^1^
α = 74.43 (3)°	*T* = 293 K
β = 81.92 (3)°	Prism, colourless
γ = 69.12 (3)°	0.26 × 0.22 × 0.20 mm
*V* = 1576.7 (7) Å^3^	

### Data collection

**Table:**

Rigaku Saturn diffractometer	6168 independent reflections
Radiation source: fine-focus sealed tube	4273 reflections with *I* > 2σ(*I*)
graphite	*R*_int_ = 0.033
Detector resolution: 7.31 pixels mm^-1^	θ_max_ = 26.0°, θ_min_ = 1.6°
ω scans	*h* = −12→12
Absorption correction: multi-scan (*CrystalClear*; Rigaku, 2008)	*k* = −16→16
*T*_min_ = 0.981, *T*_max_ = 0.985	*l* = −16→16
17336 measured reflections	

### Refinement

**Table:**

Refinement on *F*^2^	Primary atom site location: structure-invariant direct methods
Least-squares matrix: full	Secondary atom site location: difference Fourier map
*R*[*F*^2^ > 2σ(*F*^2^)] = 0.057	Hydrogen site location: inferred from neighbouring sites
*wR*(*F*^2^) = 0.174	H-atom parameters constrained
*S* = 1.06	*w* = 1/[σ^2^(*F*_o_^2^) + (0.1016*P*)^2^] where *P* = (*F*_o_^2^ + 2*F*_c_^2^)/3
6168 reflections	(Δ/σ)_max_ < 0.001
399 parameters	Δρ_max_ = 0.29 e Å^−^^3^
2 restraints	Δρ_min_ = −0.24 e Å^−^^3^

### Special details

**Table:**

Geometry. All esds (except the esd in the dihedral angle between two l.s. planes) are estimated using the full covariance matrix. The cell esds are taken into account individually in the estimation of esds in distances, angles and torsion angles; correlations between esds in cell parameters are only used when they are defined by crystal symmetry. An approximate (isotropic) treatment of cell esds is used for estimating esds involving l.s. planes.
Refinement. Refinement of *F*^2^ against ALL reflections. The weighted *R*-factor *wR* andgoodness of fit *S* are based on *F*^2^, conventional *R*-factors *R* are based on *F*, with *F* set to zero for negative *F*^2^. The threshold expression of *F*^2^ > σ(*F*^2^) is used only for calculating *R*-factors(gt) *etc*. and is not relevant to the choice of reflections for refinement. *R*-factors based on *F*^2^ are statistically about twice as large as those based on *F*, and *R*- factors based on ALL data will be even larger.

### Fractional atomic coordinates and isotropic or equivalent isotropic displacement parameters (Å^2^)

**Table:**

	*x*	*y*	*z*	*U*_iso_*/*U*_eq_	Occ. (<1)
O1	0.33256 (15)	0.41003 (11)	0.03815 (10)	0.0723 (4)	
O2	0.36695 (14)	0.00487 (9)	0.43694 (9)	0.0579 (4)	
N1	0.31962 (15)	0.25956 (11)	0.16827 (10)	0.0456 (4)	
N2	−0.00271 (18)	0.22023 (14)	0.08727 (13)	0.0650 (5)	
N3	−0.04621 (17)	0.22186 (14)	0.60535 (13)	0.0665 (5)	
N4	0.72011 (19)	−0.26509 (12)	0.27732 (12)	0.0652 (5)	
C1	0.37105 (19)	0.34525 (14)	0.12145 (13)	0.0506 (4)	
C2	0.47929 (18)	0.34194 (13)	0.18879 (13)	0.0487 (4)	
C3	0.5722 (2)	0.40412 (15)	0.17296 (16)	0.0628 (5)	
H3	0.5670	0.4626	0.1153	0.075*	
C4	0.6718 (2)	0.37619 (17)	0.24543 (18)	0.0741 (6)	
H4	0.7355	0.4163	0.2363	0.089*	
C5	0.6800 (2)	0.28913 (18)	0.33248 (17)	0.0697 (6)	
H5	0.7486	0.2719	0.3804	0.084*	
C6	0.5866 (2)	0.22835 (16)	0.34780 (13)	0.0569 (5)	
H6	0.5904	0.1708	0.4061	0.068*	
C7	0.48760 (18)	0.25501 (13)	0.27459 (12)	0.0451 (4)	
C8	0.38313 (18)	0.19473 (13)	0.26923 (11)	0.0423 (4)	
C9	0.26660 (18)	0.20220 (13)	0.35566 (11)	0.0418 (4)	
C10	0.26648 (18)	0.10992 (13)	0.43410 (12)	0.0451 (4)	
C11	0.16418 (19)	0.11548 (15)	0.51642 (13)	0.0529 (4)	
H11	0.1696	0.0512	0.5679	0.063*	
C12	0.05450 (19)	0.21522 (15)	0.52287 (13)	0.0515 (4)	
C13	0.0518 (2)	0.30963 (15)	0.44214 (13)	0.0551 (5)	
H13	−0.0214	0.3779	0.4424	0.066*	
C14	0.1558 (2)	0.30175 (14)	0.36355 (13)	0.0526 (4)	
H14	0.1523	0.3661	0.3128	0.063*	
C15	0.46646 (17)	0.07377 (13)	0.26878 (11)	0.0423 (4)	
C16	0.45792 (18)	−0.01265 (13)	0.35126 (12)	0.0446 (4)	
C17	0.54006 (19)	−0.12331 (14)	0.35451 (13)	0.0512 (4)	
H17	0.5299	−0.1786	0.4116	0.061*	
C18	0.63747 (19)	−0.15355 (14)	0.27427 (13)	0.0500 (4)	
C19	0.6473 (2)	−0.06638 (15)	0.18986 (13)	0.0585 (5)	
H19	0.7104	−0.0829	0.1341	0.070*	
C20	0.5653 (2)	0.04237 (15)	0.18886 (13)	0.0553 (5)	
H20	0.5758	0.0981	0.1323	0.066*	
C21	0.2309 (2)	0.22241 (15)	0.11800 (14)	0.0547 (5)	
H21A	0.2580	0.1416	0.1412	0.066*	
H21B	0.2539	0.2397	0.0447	0.066*	
C22	0.0696 (2)	0.27255 (18)	0.13600 (17)	0.0683 (6)	
H22A	0.0443	0.2597	0.2090	0.082*	
H22B	0.0383	0.3527	0.1070	0.082*	
C23	−0.1125 (2)	0.28245 (17)	0.03626 (15)	0.0606 (5)	
H23	−0.1429	0.3593	0.0303	0.073*	
C24	−0.1941 (2)	0.23795 (17)	−0.01401 (14)	0.0579 (5)	
C25	−0.1574 (2)	0.12469 (18)	−0.00607 (15)	0.0657 (5)	
H25	−0.0794	0.0749	0.0328	0.079*	
C26	−0.2341 (3)	0.0835 (2)	−0.05482 (16)	0.0771 (6)	
H26	−0.2064	0.0070	−0.0501	0.093*	
C27	−0.3511 (3)	0.1566 (3)	−0.10997 (18)	0.0909 (8)	
H27	−0.4045	0.1298	−0.1420	0.109*	
C28	−0.3891 (3)	0.2687 (3)	−0.1178 (2)	0.1100 (10)	
H28	−0.4688	0.3180	−0.1552	0.132*	
C29	−0.3110 (3)	0.3104 (2)	−0.07097 (19)	0.0887 (7)	
H29	−0.3373	0.3872	−0.0780	0.106*	
C30	−0.1847 (2)	0.31369 (18)	0.59963 (17)	0.0753 (6)	
H30A	−0.2600	0.2840	0.6356	0.090*	
H30B	−0.2090	0.3441	0.5283	0.090*	
C31	−0.1861 (3)	0.4074 (2)	0.6441 (2)	0.1015 (8)	
H31A	−0.2797	0.4651	0.6360	0.152*	
H31B	−0.1122	0.4378	0.6089	0.152*	
H31C	−0.1671	0.3791	0.7157	0.152*	
C32	−0.0309 (2)	0.12577 (18)	0.69285 (16)	0.0714 (6)	
H32A	−0.0778	0.1526	0.7533	0.086*	
H32B	0.0718	0.0878	0.7052	0.086*	
C33	−0.0946 (3)	0.0433 (2)	0.6787 (2)	0.1069 (9)	
H33A	−0.0736	−0.0204	0.7362	0.160*	
H33B	−0.0529	0.0194	0.6169	0.160*	
H33C	−0.1981	0.0778	0.6740	0.160*	
C34	0.8377 (3)	−0.2893 (2)	0.1969 (2)	0.0724 (8)	0.76
H34A	0.9211	−0.2707	0.2064	0.087*	0.76
H34B	0.8012	−0.2456	0.1294	0.087*	0.76
C35	0.8722 (4)	−0.4042 (3)	0.1772 (3)	0.0838 (11)	0.76
H35A	0.8985	−0.4603	0.2399	0.101*	0.76
H35B	0.9519	−0.4170	0.1271	0.101*	0.76
H35C	0.7883	−0.4083	0.1521	0.101*	0.76
C34'	0.7532 (10)	−0.3164 (7)	0.1892 (6)	0.0724 (8)	0.24
H34C	0.7230	−0.2566	0.1284	0.087*	0.24
H34D	0.6966	−0.3655	0.1967	0.087*	0.24
C35'	0.9113 (12)	−0.3765 (11)	0.1716 (13)	0.0838 (11)	0.24
H35D	0.9623	−0.3235	0.1512	0.101*	0.24
H35E	0.9241	−0.4154	0.1185	0.101*	0.24
H35F	0.9493	−0.4294	0.2338	0.101*	0.24
C36	0.7264 (2)	−0.34727 (17)	0.37557 (17)	0.0726 (6)	
H36A	0.7233	−0.3119	0.4305	0.087*	
H36B	0.8185	−0.4080	0.3772	0.087*	
C37	0.6059 (3)	−0.3945 (2)	0.3947 (2)	0.0971 (8)	
H37A	0.5141	−0.3346	0.3895	0.146*	
H37B	0.6116	−0.4430	0.4622	0.146*	
H37C	0.6141	−0.4364	0.3447	0.146*	

### Atomic displacement parameters (Å^2^)

**Table:**

	*U*^11^	*U*^22^	*U*^33^	*U*^12^	*U*^13^	*U*^23^
O1	0.0802 (10)	0.0692 (9)	0.0529 (8)	−0.0277 (8)	−0.0096 (7)	0.0163 (7)
O2	0.0672 (8)	0.0433 (7)	0.0453 (7)	−0.0118 (6)	0.0151 (6)	0.0002 (5)
N1	0.0533 (8)	0.0474 (8)	0.0364 (7)	−0.0224 (7)	−0.0069 (6)	−0.0005 (6)
N2	0.0600 (10)	0.0694 (10)	0.0723 (11)	−0.0221 (8)	−0.0126 (9)	−0.0222 (8)
N3	0.0568 (10)	0.0709 (10)	0.0628 (10)	−0.0188 (8)	0.0170 (8)	−0.0147 (8)
N4	0.0704 (11)	0.0488 (9)	0.0634 (10)	−0.0074 (8)	0.0043 (8)	−0.0128 (7)
C1	0.0550 (10)	0.0468 (9)	0.0428 (9)	−0.0187 (8)	0.0009 (8)	0.0016 (7)
C2	0.0527 (10)	0.0439 (9)	0.0494 (10)	−0.0218 (8)	0.0033 (8)	−0.0060 (7)
C3	0.0675 (12)	0.0515 (11)	0.0722 (13)	−0.0327 (10)	0.0081 (10)	−0.0077 (9)
C4	0.0715 (14)	0.0742 (14)	0.0979 (17)	−0.0446 (12)	0.0074 (12)	−0.0334 (13)
C5	0.0708 (13)	0.0849 (15)	0.0701 (13)	−0.0379 (12)	−0.0069 (10)	−0.0277 (11)
C6	0.0650 (12)	0.0646 (11)	0.0467 (10)	−0.0285 (10)	−0.0059 (9)	−0.0114 (8)
C7	0.0509 (9)	0.0464 (9)	0.0412 (9)	−0.0225 (8)	0.0015 (7)	−0.0091 (7)
C8	0.0510 (9)	0.0420 (9)	0.0347 (8)	−0.0213 (7)	−0.0037 (7)	−0.0017 (6)
C9	0.0488 (9)	0.0409 (9)	0.0370 (8)	−0.0185 (8)	−0.0020 (7)	−0.0066 (7)
C10	0.0479 (9)	0.0425 (9)	0.0430 (9)	−0.0172 (8)	0.0007 (7)	−0.0059 (7)
C11	0.0591 (11)	0.0514 (10)	0.0461 (10)	−0.0249 (9)	0.0088 (8)	−0.0054 (8)
C12	0.0502 (10)	0.0578 (11)	0.0507 (10)	−0.0227 (9)	0.0050 (8)	−0.0171 (8)
C13	0.0559 (11)	0.0497 (10)	0.0541 (10)	−0.0115 (8)	−0.0003 (8)	−0.0126 (8)
C14	0.0643 (11)	0.0449 (10)	0.0444 (9)	−0.0184 (9)	−0.0008 (8)	−0.0044 (7)
C15	0.0466 (9)	0.0437 (9)	0.0367 (8)	−0.0194 (7)	−0.0008 (7)	−0.0042 (7)
C16	0.0475 (9)	0.0474 (10)	0.0396 (8)	−0.0205 (8)	0.0034 (7)	−0.0077 (7)
C17	0.0565 (10)	0.0426 (10)	0.0482 (10)	−0.0155 (8)	0.0019 (8)	−0.0041 (7)
C18	0.0472 (9)	0.0503 (10)	0.0510 (10)	−0.0134 (8)	−0.0032 (8)	−0.0131 (8)
C19	0.0634 (12)	0.0615 (12)	0.0445 (10)	−0.0178 (10)	0.0106 (8)	−0.0137 (8)
C20	0.0623 (11)	0.0529 (11)	0.0434 (9)	−0.0189 (9)	0.0073 (8)	−0.0051 (8)
C21	0.0599 (11)	0.0590 (11)	0.0493 (10)	−0.0219 (9)	−0.0108 (8)	−0.0125 (8)
C22	0.0575 (12)	0.0759 (13)	0.0827 (14)	−0.0222 (10)	−0.0096 (10)	−0.0340 (11)
C23	0.0518 (11)	0.0662 (12)	0.0636 (12)	−0.0170 (10)	−0.0020 (9)	−0.0192 (10)
C24	0.0488 (10)	0.0715 (13)	0.0538 (11)	−0.0186 (9)	−0.0001 (8)	−0.0188 (9)
C25	0.0618 (12)	0.0814 (15)	0.0604 (12)	−0.0309 (11)	−0.0017 (9)	−0.0183 (10)
C26	0.0852 (16)	0.1079 (17)	0.0617 (13)	−0.0579 (14)	0.0115 (12)	−0.0309 (12)
C27	0.0702 (15)	0.161 (3)	0.0698 (15)	−0.0571 (17)	0.0031 (12)	−0.0519 (16)
C28	0.0725 (17)	0.159 (3)	0.098 (2)	−0.0152 (18)	−0.0340 (15)	−0.047 (2)
C29	0.0691 (14)	0.1021 (18)	0.0879 (17)	−0.0075 (13)	−0.0237 (13)	−0.0300 (14)
C30	0.0560 (12)	0.0891 (15)	0.0778 (14)	−0.0188 (11)	0.0111 (10)	−0.0295 (12)
C31	0.103 (2)	0.0996 (19)	0.0962 (18)	−0.0119 (15)	−0.0012 (15)	−0.0454 (15)
C32	0.0767 (14)	0.0844 (14)	0.0616 (12)	−0.0423 (12)	0.0242 (10)	−0.0242 (10)
C33	0.115 (2)	0.114 (2)	0.116 (2)	−0.0718 (18)	0.0348 (17)	−0.0437 (17)
C34	0.0742 (19)	0.0536 (15)	0.0874 (18)	−0.0204 (13)	0.0190 (16)	−0.0261 (13)
C35	0.098 (3)	0.072 (2)	0.0880 (19)	−0.0393 (16)	0.0252 (19)	−0.0309 (19)
C34'	0.0742 (19)	0.0536 (15)	0.0874 (18)	−0.0204 (13)	0.0190 (16)	−0.0261 (13)
C35'	0.098 (3)	0.072 (2)	0.0880 (19)	−0.0393 (16)	0.0252 (19)	−0.0309 (19)
C36	0.0678 (13)	0.0586 (12)	0.0777 (14)	−0.0027 (11)	−0.0078 (11)	−0.0169 (11)
C37	0.122 (2)	0.0702 (15)	0.0990 (19)	−0.0350 (15)	−0.0047 (17)	−0.0172 (13)

### Geometric parameters (Å, °)

**Table:**

O1—C1	1.2319 (19)	C21—H21A	0.9700
O2—C10	1.377 (2)	C21—H21B	0.9700
O2—C16	1.3810 (19)	C22—H22A	0.9700
N1—C1	1.364 (2)	C22—H22B	0.9700
N1—C21	1.455 (2)	C23—C24	1.468 (3)
N1—C8	1.491 (2)	C23—H23	0.9300
N2—C23	1.261 (2)	C24—C29	1.378 (3)
N2—C22	1.463 (2)	C24—C25	1.379 (3)
N3—C12	1.385 (2)	C25—C26	1.387 (3)
N3—C30	1.459 (3)	C25—H25	0.9300
N3—C32	1.461 (3)	C26—C27	1.370 (3)
N4—C18	1.393 (2)	C26—H26	0.9300
N4—C36	1.466 (3)	C27—C28	1.364 (4)
N4—C34	1.482 (3)	C27—H27	0.9300
N4—C34'	1.467 (7)	C28—C29	1.386 (3)
C1—C2	1.479 (2)	C28—H28	0.9300
C2—C7	1.383 (2)	C29—H29	0.9300
C2—C3	1.391 (2)	C30—C31	1.506 (3)
C3—C4	1.371 (3)	C30—H30A	0.9700
C3—H3	0.9300	C30—H30B	0.9700
C4—C5	1.395 (3)	C31—H31A	0.9600
C4—H4	0.9300	C31—H31B	0.9600
C5—C6	1.381 (3)	C31—H31C	0.9600
C5—H5	0.9300	C32—C33	1.496 (3)
C6—C7	1.378 (2)	C32—H32A	0.9700
C6—H6	0.9300	C32—H32B	0.9700
C7—C8	1.524 (2)	C33—H33A	0.9600
C8—C15	1.509 (2)	C33—H33B	0.9600
C8—C9	1.517 (2)	C33—H33C	0.9600
C9—C10	1.382 (2)	C34—C35	1.515 (4)
C9—C14	1.393 (2)	C34—H34A	0.9700
C10—C11	1.393 (2)	C34—H34B	0.9801
C11—C12	1.385 (3)	C35—H35A	0.9600
C11—H11	0.9300	C35—H35B	0.9600
C12—C13	1.412 (2)	C35—H35C	0.9600
C13—C14	1.367 (2)	C34'—C35'	1.492 (9)
C13—H13	0.9300	C34'—H34C	0.9696
C14—H14	0.9300	C34'—H34D	0.9710
C15—C16	1.381 (2)	C35'—H35D	0.9600
C15—C20	1.395 (2)	C35'—H35E	0.9600
C16—C17	1.382 (2)	C35'—H35F	0.9600
C17—C18	1.388 (2)	C36—C37	1.488 (3)
C17—H17	0.9300	C36—H36A	0.9700
C18—C19	1.406 (2)	C36—H36B	0.9700
C19—C20	1.366 (2)	C37—H37A	0.9600
C19—H19	0.9300	C37—H37B	0.9600
C20—H20	0.9300	C37—H37C	0.9600
C21—C22	1.498 (3)		
			
C10—O2—C16	118.54 (12)	N2—C22—C21	108.56 (16)
C1—N1—C21	123.41 (14)	N2—C22—H22A	110.0
C1—N1—C8	113.61 (13)	C21—C22—H22A	110.0
C21—N1—C8	122.21 (13)	N2—C22—H22B	110.0
C23—N2—C22	118.20 (18)	C21—C22—H22B	110.0
C12—N3—C30	121.57 (16)	H22A—C22—H22B	108.4
C12—N3—C32	120.37 (16)	N2—C23—C24	122.38 (19)
C30—N3—C32	116.85 (16)	N2—C23—H23	118.8
C18—N4—C36	118.08 (16)	C24—C23—H23	118.8
C18—N4—C34	118.14 (17)	C29—C24—C25	118.5 (2)
C36—N4—C34	119.76 (17)	C29—C24—C23	119.7 (2)
C18—N4—C34'	124.8 (4)	C25—C24—C23	121.83 (18)
C36—N4—C34'	112.6 (4)	C24—C25—C26	121.4 (2)
O1—C1—N1	124.55 (17)	C24—C25—H25	119.3
O1—C1—C2	128.61 (16)	C26—C25—H25	119.3
N1—C1—C2	106.83 (13)	C27—C26—C25	119.3 (2)
C7—C2—C3	120.98 (17)	C27—C26—H26	120.3
C7—C2—C1	108.63 (14)	C25—C26—H26	120.3
C3—C2—C1	130.17 (16)	C28—C27—C26	119.8 (2)
C4—C3—C2	117.64 (17)	C28—C27—H27	120.1
C4—C3—H3	121.2	C26—C27—H27	120.1
C2—C3—H3	121.2	C27—C28—C29	121.0 (2)
C3—C4—C5	121.62 (18)	C27—C28—H28	119.5
C3—C4—H4	119.2	C29—C28—H28	119.5
C5—C4—H4	119.2	C24—C29—C28	119.9 (3)
C6—C5—C4	120.29 (19)	C24—C29—H29	120.0
C6—C5—H5	119.9	C28—C29—H29	120.0
C4—C5—H5	119.9	N3—C30—C31	114.5 (2)
C7—C6—C5	118.37 (17)	N3—C30—H30A	108.6
C7—C6—H6	120.8	C31—C30—H30A	108.6
C5—C6—H6	120.8	N3—C30—H30B	108.6
C6—C7—C2	121.08 (16)	C31—C30—H30B	108.6
C6—C7—C8	128.26 (14)	H30A—C30—H30B	107.6
C2—C7—C8	110.54 (14)	C30—C31—H31A	109.5
N1—C8—C15	111.68 (13)	C30—C31—H31B	109.5
N1—C8—C9	111.33 (13)	H31A—C31—H31B	109.5
C15—C8—C9	110.18 (12)	C30—C31—H31C	109.5
N1—C8—C7	100.36 (12)	H31A—C31—H31C	109.5
C15—C8—C7	110.20 (13)	H31B—C31—H31C	109.5
C9—C8—C7	112.79 (13)	N3—C32—C33	113.9 (2)
C10—C9—C14	115.44 (15)	N3—C32—H32A	108.8
C10—C9—C8	121.58 (14)	C33—C32—H32A	108.8
C14—C9—C8	122.95 (13)	N3—C32—H32B	108.8
O2—C10—C9	122.77 (15)	C33—C32—H32B	108.8
O2—C10—C11	114.51 (14)	H32A—C32—H32B	107.7
C9—C10—C11	122.72 (16)	C32—C33—H33A	109.5
C12—C11—C10	121.02 (16)	C32—C33—H33B	109.5
C12—C11—H11	119.5	H33A—C33—H33B	109.5
C10—C11—H11	119.5	C32—C33—H33C	109.5
N3—C12—C11	121.37 (16)	H33A—C33—H33C	109.5
N3—C12—C13	121.87 (16)	H33B—C33—H33C	109.5
C11—C12—C13	116.76 (16)	N4—C34—C35	112.9 (2)
C14—C13—C12	120.69 (16)	N4—C34—H34A	112.6
C14—C13—H13	119.7	C35—C34—H34A	115.5
C12—C13—H13	119.7	N4—C34—H34B	109.8
C13—C14—C9	123.34 (15)	C35—C34—H34B	97.1
C13—C14—H14	118.3	H34A—C34—H34B	107.5
C9—C14—H14	118.3	N4—C34'—C35'	113.8 (9)
C16—C15—C20	115.59 (15)	N4—C34'—H34C	107.5
C16—C15—C8	121.89 (14)	C35'—C34'—H34C	106.6
C20—C15—C8	122.34 (14)	N4—C34'—H34D	109.6
O2—C16—C15	122.60 (15)	C35'—C34'—H34D	111.1
O2—C16—C17	114.83 (14)	H34B—C34'—H34D	146.2
C15—C16—C17	122.57 (16)	H34C—C34'—H34D	107.9
C16—C17—C18	121.29 (15)	C34'—C35'—H35D	109.5
C16—C17—H17	119.4	C34'—C35'—H35E	109.5
C18—C17—H17	119.4	H35D—C35'—H35E	109.5
C17—C18—N4	121.37 (16)	C34'—C35'—H35F	109.5
C17—C18—C19	116.72 (16)	H35D—C35'—H35F	109.5
N4—C18—C19	121.91 (16)	H35E—C35'—H35F	109.5
C20—C19—C18	120.76 (16)	N4—C36—C37	113.50 (19)
C20—C19—H19	119.6	N4—C36—H36A	108.9
C18—C19—H19	119.6	C37—C36—H36A	108.9
C19—C20—C15	123.05 (16)	N4—C36—H36B	108.9
C19—C20—H20	118.5	C37—C36—H36B	108.9
C15—C20—H20	118.5	H36A—C36—H36B	107.7
N1—C21—C22	115.50 (15)	C36—C37—H37A	109.5
N1—C21—H21A	108.4	C36—C37—H37B	109.5
C22—C21—H21A	108.4	H37A—C37—H37B	109.5
N1—C21—H21B	108.4	C36—C37—H37C	109.5
C22—C21—H21B	108.4	H37A—C37—H37C	109.5
H21A—C21—H21B	107.5	H37B—C37—H37C	109.5
			
C21—N1—C1—O1	10.6 (3)	N1—C8—C15—C16	−138.91 (15)
C8—N1—C1—O1	−179.33 (17)	C9—C8—C15—C16	−14.6 (2)
C21—N1—C1—C2	−168.62 (15)	C7—C8—C15—C16	110.44 (17)
C8—N1—C1—C2	1.50 (19)	N1—C8—C15—C20	46.1 (2)
O1—C1—C2—C7	−179.92 (18)	C9—C8—C15—C20	170.38 (15)
N1—C1—C2—C7	−0.79 (19)	C7—C8—C15—C20	−64.54 (19)
O1—C1—C2—C3	−5.3 (3)	C10—O2—C16—C15	9.4 (2)
N1—C1—C2—C3	173.79 (18)	C10—O2—C16—C17	−170.81 (14)
C7—C2—C3—C4	−0.1 (3)	C20—C15—C16—O2	179.04 (15)
C1—C2—C3—C4	−174.11 (18)	C8—C15—C16—O2	3.7 (2)
C2—C3—C4—C5	−0.4 (3)	C20—C15—C16—C17	−0.8 (2)
C3—C4—C5—C6	0.0 (3)	C8—C15—C16—C17	−176.08 (15)
C4—C5—C6—C7	0.9 (3)	O2—C16—C17—C18	−179.34 (15)
C5—C6—C7—C2	−1.4 (3)	C15—C16—C17—C18	0.5 (3)
C5—C6—C7—C8	174.27 (18)	C16—C17—C18—N4	−179.84 (16)
C3—C2—C7—C6	1.0 (3)	C16—C17—C18—C19	−0.4 (3)
C1—C2—C7—C6	176.16 (16)	C36—N4—C18—C17	−13.2 (3)
C3—C2—C7—C8	−175.35 (16)	C34—N4—C18—C17	−170.6 (2)
C1—C2—C7—C8	−0.18 (19)	C34'—N4—C18—C17	141.0 (5)
C1—N1—C8—C15	−118.30 (15)	C36—N4—C18—C19	167.39 (18)
C21—N1—C8—C15	52.0 (2)	C34—N4—C18—C19	10.0 (3)
C1—N1—C8—C9	118.08 (15)	C34'—N4—C18—C19	−38.4 (5)
C21—N1—C8—C9	−71.67 (19)	C17—C18—C19—C20	0.7 (3)
C1—N1—C8—C7	−1.53 (17)	N4—C18—C19—C20	−179.88 (18)
C21—N1—C8—C7	168.72 (15)	C18—C19—C20—C15	−1.1 (3)
C6—C7—C8—N1	−175.03 (17)	C16—C15—C20—C19	1.1 (3)
C2—C7—C8—N1	0.97 (17)	C8—C15—C20—C19	176.36 (16)
C6—C7—C8—C15	−57.2 (2)	C1—N1—C21—C22	−94.8 (2)
C2—C7—C8—C15	118.84 (15)	C8—N1—C21—C22	95.9 (2)
C6—C7—C8—C9	66.4 (2)	C23—N2—C22—C21	−135.87 (19)
C2—C7—C8—C9	−117.58 (15)	N1—C21—C22—N2	−175.59 (14)
N1—C8—C9—C10	138.75 (15)	C22—N2—C23—C24	−178.91 (17)
C15—C8—C9—C10	14.3 (2)	N2—C23—C24—C29	−178.3 (2)
C7—C8—C9—C10	−109.32 (17)	N2—C23—C24—C25	1.7 (3)
N1—C8—C9—C14	−43.2 (2)	C29—C24—C25—C26	0.7 (3)
C15—C8—C9—C14	−167.67 (14)	C23—C24—C25—C26	−179.25 (17)
C7—C8—C9—C14	68.73 (19)	C24—C25—C26—C27	−1.6 (3)
C16—O2—C10—C9	−9.7 (2)	C25—C26—C27—C28	1.1 (4)
C16—O2—C10—C11	169.93 (14)	C26—C27—C28—C29	0.2 (4)
C14—C9—C10—O2	178.82 (15)	C25—C24—C29—C28	0.6 (3)
C8—C9—C10—O2	−3.0 (2)	C23—C24—C29—C28	−179.4 (2)
C14—C9—C10—C11	−0.8 (2)	C27—C28—C29—C24	−1.0 (4)
C8—C9—C10—C11	177.40 (15)	C12—N3—C30—C31	−97.3 (2)
O2—C10—C11—C12	−178.66 (15)	C32—N3—C30—C31	95.2 (2)
C9—C10—C11—C12	1.0 (3)	C12—N3—C32—C33	−84.8 (2)
C30—N3—C12—C11	−161.22 (18)	C30—N3—C32—C33	82.9 (2)
C32—N3—C12—C11	5.9 (3)	C18—N4—C34—C35	−153.8 (3)
C30—N3—C12—C13	19.7 (3)	C36—N4—C34—C35	49.1 (4)
C32—N3—C12—C13	−173.26 (17)	C34'—N4—C34—C35	−42.0 (6)
C10—C11—C12—N3	−178.91 (17)	C18—N4—C34'—C35'	128.0 (7)
C10—C11—C12—C13	0.3 (3)	C36—N4—C34'—C35'	−76.5 (9)
N3—C12—C13—C14	177.52 (17)	C34—N4—C34'—C35'	33.5 (7)
C11—C12—C13—C14	−1.6 (3)	C18—N4—C36—C37	87.3 (2)
C12—C13—C14—C9	1.9 (3)	C34—N4—C36—C37	−115.6 (2)
C10—C9—C14—C13	−0.7 (3)	C34'—N4—C36—C37	−69.9 (5)
C8—C9—C14—C13	−178.82 (16)		
